# *Clostridium butyricum* GKB7 increase Physical Performance and Improve Biochemistry Profile on Mice with Inherently Low Aerobic Capacity

**DOI:** 10.7150/ijms.118805

**Published:** 2025-10-24

**Authors:** Mon-Chien Lee, Chao-Yuan Chen, Yi-Ju Hsu, Shih-Wei Lin, You-Shan Tsai, Yen-Lien Chen, Chin-Chu Chen, Chi-Chang Huang

**Affiliations:** 1 Graduate Institute of Sports Science, National Taiwan Sport University, Taoyuan City 333325, Taiwan.; 2 Center for General Education, Taipei Medical University, Taipei 110301, Taiwan.; 3 Physical Education Office, National Taipei University of Business, Taipei, Taiwan.; 4 Biotech Research Institute, Grape King Bio Ltd., Taoyuan City 325002, Taiwan.

**Keywords:** GKB7, exercise performance, glycogen storage, gut microbiota, aerobic endurance

## Abstract

**Introduction:** This study investigates the effects of *Clostridium butyricum* GKB7 (CB), a product manufactured by Grape King Bio Ltd., on enhancing exercise performance and blood biochemistry in mice with intrinsically low aerobic exercise capacity.

**Methods:** Using a low aerobic exercise capacity mouse model (n = 48), animals were divided into six groups (n = 8 per group) based on body weight balancing principles: Control group (Vehicle), Positive control group (BCAA), Low-dose CB group (CB-L; 0.01 g human equivalent dose), High-dose CB group (CB-H; 0.1 g human equivalent dose), Low-dose heat-killed CB group (HK-CB-L; 0.01 g human equivalent dose), and High-dose heat-killed CB group (HK-CB-H; 0.1 g human equivalent dose). After four weeks of continuous supplementation, aerobic endurance and balance-related tests were performed, including weight-loaded swimming to exhaustion, treadmill running to exhaustion, and rotarod running, along with oral glucose tolerance test (OGTT) assessments. The experimental protocol involved daily supplementation of CB in different dosages, followed by endurance performance assessments and biochemical analyses.

**Results:** We evaluated the effects of four-week supplementation with live or heat-killed *Clostridium butyricum* GKB7 (CB; low/high dose) on performance and energy stores in mice with low innate exercise capacity. Both live and heat-killed CB improved aerobic endurance versus vehicle: weight-loaded swimming time increased 1.49-1.87-fold, and treadmill running time increased 1.16-1.22-fold (all *p*<0.05 vs vehicle; BCAA: 1.09-fold vs vehicle; no significant CB-BCAA differences on treadmill). Neuromotor performance also improved: maximum and average balance times increased 1.49-2.13-fold and 1.61-2.29-fold versus vehicle (*p*<0.05); heat-killed CB groups were higher than BCAA on balance (≈1.20-1.43-fold for maximum; 1.24-1.47-fold for average). CB elevated energy reserves versus vehicle, with hepatic glycogen increased 2.55-2.74-fold and skeletal muscle glycogen increased 1.18-1.24-fold (*p*<0.05). Additionally, both live and heat-killed CB increased gut microbiota diversity and enriched symbiotic beneficial taxa. Alpha diversity increased in HK-CB-L vs. CB-L/CB-H and BCAA (*p*<0.05); F/B ratio was lower in CB-L and CB-H vs. their heat-killed counterparts (*p*<0.05). LEfSe identified taxa enriched toward dominant/symbiotic beneficial bacteria. Collectively, CB—viable or heat-killed—enhances aerobic endurance, balance performance, and glycogen storage, with heat-killed CB showing advantages over BCAA on balance.

**Conclusions:** Four-week supplementation of *C. butyricum* GKB7, significantly enhanced gut microbiota diversity and symbiotic bacterial proportions in mice with naturally low exercise capacity. Moreover, supplementation significantly improved aerobic endurance, balance performance, and glycogen storage, demonstrating its potential as an ergogenic aid.

## Introduction

Aerobic exercise capacity is a key determinant of endurance performance and overall metabolic health. However, individuals with inherently low aerobic capacity often exhibit reduced exercise performance, impaired glucose metabolism, and suboptimal physiological adaptations to physical training 1. Recent studies have suggested that gut microbiota play a crucial role in host metabolism, energy utilization, and exercise performance, highlighting the potential benefits of probiotic interventions in enhancing endurance capacity and physiological resilience 2. Studies in lines selectively bred for divergent exercise capacities indicate that low-capacity mice show metabolic abnormalities and mitochondrial impairment, and are more prone to performance fatigability predominantly mediated by peripheral mechanisms (e.g., greater metabolite accumulation and slower metabolic recovery) 3, 4. Additionally, these low-capacity mice tend to have altered muscle composition, reduced glycogen storage, and a less efficient energy metabolism, which may limit their endurance performance 5.

*Clostridium butyricum* GKB7 (CB) is a butyrate-producing anaerobic bacterium isolated from the feces of healthy Taiwanese individuals. Previous studies have demonstrated its beneficial effects on gut microbiota modulation, anti-inflammatory properties, and metabolic health improvement 6, 7. Specifically, CB supplementation has been shown to ameliorate osteoarthritis progression by reducing inflammatory cytokines and preserving cartilage integrity in rodent models 8. Additionally, CB's probiotic properties extend beyond joint health, as butyrate production has been linked to enhanced mitochondrial function, reduced oxidative stress, and improved metabolic flexibility 9, all of which are essential for sustaining endurance performance. Despite the growing body of evidence supporting the role of probiotics in exercise physiology, the specific effects of live and heat-killed *C. butyricum* GKB7 supplementation on endurance performance, balance, glycogen storage, glucose metabolism, and gut microbiota composition remain largely unexplored. Given the fundamental role of energy metabolism in endurance exercise, investigating the impact of CB supplementation on glycogen storage in liver and muscle tissues is particularly relevant.

This study aims to evaluate the ergogenic and metabolic effects of CB supplementation in mice with inherently low aerobic capacity. The key objectives include: Examining the effects of CB supplementation on aerobic endurance performance, assessed by weight-loaded swimming and treadmill running to exhaustion. Evaluating the impact of CB supplementation on coordination and balance using a rotarod test. Investigating the effects of CB supplementation on glycogen storage in liver and skeletal muscle. Assessing the role of CB supplementation in glucose metabolism through an oral glucose tolerance test (OGTT). Analyzing the influence of CB supplementation on gut microbiota diversity and composition. In contrast to many probiotic-exercise studies that assessed performance without sequencing-based microbiome profiling, we applied 16S rRNA amplicon sequencing (V3-V4) with LEfSe to resolve taxonomic shifts accompanying performance and glycogen phenotypes. Libraries were quantified by qPCR prior to Illumina sequencing, enabling robust community-level comparisons. This is the first study to evaluate a strain-specific *Clostridium butyricum* GKB7 intervention in both live and heat-killed forms across two doses in a low-innate aerobic capacity mouse model, integrating endurance, neuromotor balance, and liver/muscle glycogen outcomes. We further relate these phenotypes to 16S rRNA (V3-V4)-based community shifts (LEfSe) and benchmark effects against a BCAA positive control, thereby positioning GKB7 within established ergogenic comparators and revealing probiotic and postbiotic potential.

## Materials and Methods

### Materials

*Clostridium butyricum* GKB7 (CB) was originally isolated from the feces of healthy Taiwanese individuals, as described in a previous study 6. The bacterial strain was cultured in reinforced clostridial medium (RCM; Merck, Darmstadt, Germany) under anaerobic conditions (80% N₂, 10% H₂, and 10% CO₂) at 37 °C for 24 h. For large-scale production, 0.1% of the seed culture was inoculated into a 50-L bioreactor containing a culture medium supplemented with 5% glucose, 1.5% yeast extract, 0.5% peptone, 0.1% MgSO₄, 0.2% K₂HPO₄, 0.1% sodium acetate, and 0.2% NaCl. After 24 h of fermentation, the bacterial spores were harvested via centrifugation, followed by two washes with reverse osmosis (RO) water. The resulting pellets were freeze-dried and stored at -20 °C for subsequent use. The final lyophilized powder contained approximately **5.5 × 10⁷ CFU/g** bacterial spores.

For experimental administration, the freeze-dried ***C. butyricum* GKB7** powder was used in two forms: live bacteria (**CB**) and heat-killed bacteria (**HK-CB**). The **HK-CB** preparation involved subjecting washed bacterial pellets to heat treatment at 121 °C for 15 min, followed by freeze-drying 7. The resulting heat-killed powder was used in the study. Both formulations were administered to animals at dosages of 100 mg/kg (high dose) and 10 mg/kg (low dose) based on body weight.

### Experimental Design

Male mice with a genetic predisposition for low innate aerobic exercise capacity were used in this study. All mice were 8 weeks old at the start of the experiment and were bred in-house at the Animal Facility of the Institute of Sports Science, National Taiwan Sport University (Taoyuan, Taiwan). The mice had an initial body weight of approximately 30 g and were housed under controlled conditions with a temperature of 24 ± 2 °C, humidity of 65 ± 5%, and a 12:12 h light-dark cycle. Standard chow diet (No. 5001, PMI Nutrition International, Brentwood, MO, USA) and water were provided *ad libitum*. The animal experiment protocol was approved by the Institutional Animal Care and Use Committee (IACUC) of National Taiwan Sport University (approval number: IACUC-11006).

Experimental grouping and supplementation: After an initial 2-week acclimation period, mice were randomly assigned into six experimental groups (n = 8 per group): Vehicle group: received a standard chow diet and vehicle solution. Positive control group: supplemented with branched-chain amino acids (BCAA). Low-dose *C. butyricum* GKB7 group (CB-L): received 0.01 g of C. butyricum GKB7 (CB), corresponding to the human equivalent dose. High-dose *C. butyricum* GKB7 group (CB-H): received 0.1 g of CB, corresponding to the human equivalent dose. Low-dose heat-killed *C. butyricum* GKB7 group (HK-CB-L): supplemented with low-dose (0.01 g) heat-killed CB. High-dose heat-killed *C. butyricum* GKB7 group (HK-CB-H): supplemented with high-dose (0.1 g) heat-killed CB.

Experimental procedure: The animal trial lasted 4 weeks, during which mice were administered the test supplements daily. Mice in the treatment groups received either live CB or heat-killed CB for the entire supplementation period. Body weight, food intake, and water consumption were recorded weekly. To ensure adherence to animal welfare principles, the exercise challenge was designed with progressive intensity, starting from low intensity and short duration, then gradually increasing to higher intensity and longer duration. After 4 weeks of supplementation, all mice underwent exercise performance assessments and fatigue-related biochemical analyses. Blood samples were collected at designated time points for subsequent analysis of physiological and biochemical markers.

### Evaluation of Endurance Performance via Weight-Bearing Swimming to Exhaustion

To assess the potential endurance-enhancing effects of a four-week supplementation with either live or heat-killed *C. butyricum* GKB7 (CB), a weight-bearing swimming-to-exhaustion test was performed. Swimming adaptation: One week prior to the experiment, all mice underwent a swimming adaptation period to acclimate to the testing conditions. Adaptation sessions were conducted in a cylindrical water tank (diameter: 28 cm, water depth: 25 cm) maintained at a temperature of 27 ± 1 °C. Experimental protocol: On day 29 of the experiment, a weight-bearing swimming test was conducted to evaluate endurance performance. Mice were fasted for 12 hours before the test and received their respective supplementation 30 minutes prior to the swimming trial. Each mouse was individually placed into the water tank under forced swimming conditions, with a weight equivalent to 5% of body weight attached to its tail. Throughout the test, water temperature was maintained at 27 ± 1 °C. To ensure continuous movement, mice were required to actively swim throughout the test duration. If a mouse remained immobile and floated on the water surface, a stirring rod was used to gently agitate the water nearby to encourage movement. The time to exhaustion was recorded as the duration from the start of the swimming test until the mouse was fully submerged for 8 consecutive seconds without resurfacing 10.

### Evaluation of Endurance Performance via Treadmill Running to Exhaustion

To assess the effects of a four-week supplementation with either live or heat-killed *C. butyricum* GKB7 (CB) on endurance capacity, a treadmill running-to-exhaustion test was conducted on day 31 of the experiment. Treadmill running protocol: Endurance performance was evaluated using a rodent-specific treadmill (MK-680C, Murmachi Kikai Co. Ltd., Japan). Mice were subjected to a progressive workload protocol with an initial running speed of 12 m/min, which was increased by 3 m/min every 2 minutes until exhaustion. The treadmill was set at a 15-degree incline throughout the test. To ensure continuous running, an electrically stimulated zone was activated at the rear of the treadmill, delivering 2 Hz pulses at 1.22 mA to encourage movement. Mice were deemed exhausted when they remained in the shock zone for 5 consecutive seconds without attempting to resume running. The total running time until exhaustion was recorded as an indicator of endurance performance 11.

### Evaluation of Coordination and Balance Performance via Rotarod Test

To assess the effects of a four-week supplementation with either live or heat-killed *C. butyricum* GKB7 (CB) on coordination and balance, a rotarod test was performed. Rotarod test protocol: A rodent rotarod apparatus was used to evaluate motor coordination and balance. Each mouse was placed on a rotating cylindrical drum, with the initial speed set at 4 rpm. The rotational speed was then gradually increased to 40 rpm over a 5-minute period. The latency to fall was recorded, defined as the time (in seconds) until the mouse fell off the rotating rod. Additionally, the rotational speed at which the mouse lost balance and fell (measured in rpm) was documented as an indicator of motor coordination and balance performance 12.

### Evaluation of Glucose Tolerance via Oral Glucose Tolerance Test (OGTT)

To assess the effects of a four-week supplementation with either live or heat-killed *C. butyricum* GKB7 (CB) on glucose tolerance, an oral glucose tolerance test (OGTT) was conducted. Preparation of glucose solution: A 25% glucose solution was prepared by dissolving 3.75 g of glucose powder in 15 mL of deionized water. Each mouse received an oral glucose load equivalent to 2.5 g/kg body weight (BW), with the volume of administration calculated as 10 times the individual body weight. For example, a 30 g mouse received 0.3 mL of the 25% glucose solution. Glucose tolerance test protocol: To ensure accurate blood glucose measurements, each mouse was administered glucose at 1-minute intervals to allow adequate time for sample collection. Baseline (0 min) blood glucose levels were measured immediately before glucose administration, followed by subsequent measurements at 15, 30, 60, and 120 minutes post-administration to assess glucose tolerance. Blood glucose measurement: Blood samples were collected from the tail vein, and blood glucose levels were immediately analyzed using a glucometer (AccuChek®, Germany) at each time point 1. After the completion of the OGTT, food and water were returned to the cages to terminate the fasting period.

### Evaluation of Blood Biochemical Parameters

To assess the effects of a four-week supplementation with either live or heat-killed *C. butyricum* GKB7 (CB) on blood biochemical markers, blood samples were collected for analysis following the protocol described in the endurance performance experiment. Blood sample collection: Blood samples were obtained via cardiac puncture following euthanasia under anesthesia. The collected blood was immediately centrifuged at 3,000 × g for 10 min at 4°C to separate the serum, which was then stored at -80°C until further analysis. Biochemical analysis: The following biochemical parameters were analyzed using an automated clinical chemistry analyzer: (1) Liver function markers: Aspartate aminotransferase (AST) and alanine aminotransferase (ALT); (2) Muscle damage marker: Creatine kinase (CK); (3) Renal function markers: Blood urea nitrogen (BUN), creatinine (CREA), and uric acid (UA); (4) Lipid profile markers: Total cholesterol (TC), triglycerides (TG), high-density lipoprotein cholesterol (HDL), and low-density lipoprotein cholesterol (LDL); (5) Glucose metabolism marker: Glucose (GLU). All biochemical assays were performed using commercially available enzymatic kits according to the manufacturer's instructions.

### Evaluation of Hepatic and Skeletal Muscle Glycogen Content

To assess the effects of a four-week supplementation with either live or heat-killed *C. butyricum* GKB7 (CB) on glycogen storage in the liver and skeletal muscle, glycogen content was quantified using a chemical assay based on previously described methods 13. Previous studies often used the method of collecting organs 30 minutes after eating to observe the key window of acute postprandial metabolism 14, 15. In this study, all mice were sacrificed 30 minutes after the final supplementation, and liver and hind limb gastrocnemius muscle tissues were harvested. The collected tissues were rinsed with physiological saline, blotted dry, and weighed. Tissue samples were excised from the same anatomical location in each animal and stored at -80 °C for subsequent glycogen analysis. Glycogen extraction and quantification: For glycogen extraction, frozen tissue samples were homogenized in 5 volumes (w/v) of homogenization buffer using a Bullet Blender tissue homogenizer. The homogenate was aliquoted into microcentrifuge tubes and centrifuged at 12,000 × g for 15 minutes at 4 °C. The supernatant was collected and used for glycogen quantification. Glycogen assay: Glycogen content in liver and muscle tissues was quantified using a chemical assay. A standard calibration curve was generated using commercial glycogen standards (Sigma-Aldrich, USA) to determine glycogen concentrations in tissue samples. The results were expressed as glycogen content per gram of tissue, and the differences in glycogen storage among experimental groups were analyzed.

### Evaluation of Gut Microbiota Composition via 16S rRNA Sequencing

To investigate the effects of four-week supplementation with either live or heat-killed Clostridium butyricum GKB7 (CB) on gut microbiota composition, 16S rRNA sequencing was performed on cecal microbiota samples. Sample collection and DNA extraction: At the time of sacrifice, cecal contents were collected from a representative subset of four mice per group and immediately stored at -80 °C until further analysis. Bacterial genomic DNA was extracted from the fecal samples using the Qiagen DNA Mini Kit (Qiagen, MD, USA) following the manufacturer's protocol. The purity and concentration of the extracted DNA were assessed using a NanoDrop ND-1000 spectrophotometer (Thermo Scientific, Wilmington, DE, USA), and DNA samples were stored at -80 °C for further analysis. 16S rRNA amplicon library preparation and sequencing: The V3-V4 hypervariable region of the 16S rRNA gene was amplified via polymerase chain reaction (PCR) using the universal barcode primers 341F and 805R:

Forward primer (341F): 5'-TCGTCGGCAGCGTCAGATGTGTATAAGAGACAGCTACGGGNGGCWGCAG-3'

Reverse primer (805R): 5'-GTCTCGTGGGCTCGGAGATGTGTATAAGAGACAGGACTACHVGGGTATCTAATCC-3'

The resulting PCR amplicons were purified using AMPure XP beads (Beckman Coulter, USA). A second PCR reaction was performed to attach dual indices and Illumina sequencing adapters using primers from the Nextera XT Index Kit (Illumina, San Diego, CA, USA). Following the second PCR reaction, the final sequencing library (~630 bp) was purified again using AMPure XP beads. The concentration of the 16S rRNA sequencing library was quantified using the KAPA Library Quantification Kit (KAPA Biosystems, USA) with Illumina adapter-specific primers through real-time quantitative PCR (qPCR). The prepared amplicon libraries were denatured and sequenced using the Illumina HiSeq platform with paired-end (2 × 300 bp) sequencing chemistry (Reagent v3). Cluster generation, imaging, and base calling were performed using the HiSeq real-time analysis software (RTA), HiSeq control software (MCS), and HiSeq Report software. The sequencing output in FASTQ format was used for downstream bioinformatics analysis.

Taxonomic profiling and data analysis: Taxonomic classification of fecal microbiota was performed using 16S Metagenomics v1.0 on the HiSeq platform, targeting the V3-V4 regions of the 16S rRNA gene. Bacterial taxonomic classification was conducted based on the Greengenes database (http://greengenes.lbl.gov/), allowing for microbial community profiling at different taxonomic levels.

### Statistical Analysis

All data are presented as mean ± standard deviation (SD). Each experimental group consisted of 8 mice (n = 8 per group). Statistical analyses were performed using the SAS statistical software package (SAS Institute, Cary, NC, USA). Differences between groups were analyzed using one-way analysis of variance (ANOVA). A p-value < 0.05 was considered statistically significant.

## Results

### Body Weight and Food Intake Following Supplementation with Live or Heat-Killed *C. butyricum* GKB7

The changes in body weight, water intake, and food consumption during the experimental period for each group are summarized in **Table 1**. Throughout the four-week supplementation period, the body weight of all six groups showed a steady and progressive increase over time, indicating that neither low-dose nor high-dose supplementation of live or heat-killed *C. butyricum* GKB7 (CB) induced any adverse effects on body weight. Additionally, no significant differences were observed in initial or final body weight among the six experimental groups. Furthermore,** Table 1** shows that daily food intake remained consistent across all groups throughout the experimental period. There were no significant differences in mean daily food intake among the six groups, suggesting that four weeks of supplementation with either live or heat-killed CB at different doses did not significantly impact food or water intake.

### Supplementation with live or heat-killed *C. butyricum* GKB7 Improves Aerobic Endurance in Mice with Low Innate Exercise Capacity

To evaluate whether supplementation with low or high doses of live *C. butyricum* GKB7 (CB) or heat-killed CB enhances aerobic endurance, mice in all six experimental groups underwent swimming endurance and treadmill running tests after four weeks of supplementation. The aerobic endurance performance of each group was recorded.

**Figure 1:**
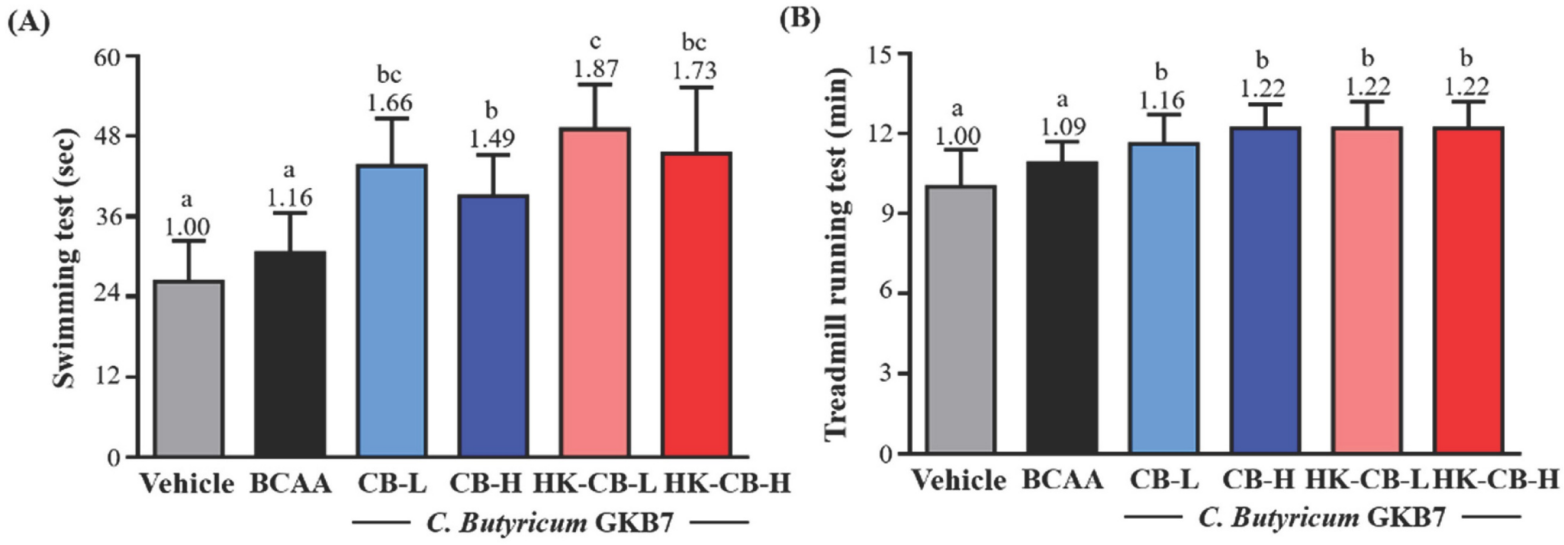
Effects of live or heat-killed *C. butyricum* GKB7 supplementation on aerobic exercise performance. **(A)** Swimming endurance and **(B)** treadmill endurance performance in each experimental group. The experimental animals were divided into six groups (n = 8 per group): Vehicle control group (Vehicle); Branched-chain amino acid control group (BCAA); Low-dose *C. butyricum* GKB7 group (CB-L); High-dose *C. butyricum* GKB7 group (CB-H); Low-dose heat-killed *C. butyricum* GKB7 group (HK-CB-L); High-dose heat-killed *C. butyricum* GKB7 group (HK-CB-H). All values are expressed as Mean ± SD. Different letters (a, b, c) indicate significant differences among groups, *P* < 0.05.

Swimming endurance performance: As shown in **Figure 1A**, the swimming endurance times for the Vehicle, BCAA, CB-L, CB-H, HK-CB-L, and HK-CB-H groups were 26.3 ± 6.1, 30.5 ± 6.0, 43.6 ± 7.1, 39.0 ± 6.2, 49.1 ± 6.7, and 45.4 ± 9.9 seconds, respectively. Compared to the Vehicle group, CB-L, CB-H, HK-CB-L, and HK-CB-H supplementation significantly increased swimming endurance by 1.66-fold (*p* < 0.0001), 1.49-fold (*p* = 0.0009), 1.87-fold (*p* < 0.0001), and 1.73-fold (*p* < 0.0001), respectively. Compared to the BCAA group, CB-L, CB-H, HK-CB-L, and HK-CB-H supplementation significantly increased swimming endurance by 1.43-fold (*p* = 0.0007), 1.28-fold (*p* = 0.0218), 1.87-fold (*p* < 0.0001), and 1.73-fold (*p* = 0.0001), respectively.

Treadmill endurance performance: The treadmill endurance times for the Vehicle, BCAA, CB-L, CB-H, HK-CB-L, and HK-CB-H groups were 10.0 ± 1.4, 10.9 ± 0.8, 11.6 ± 1.1, 12.2 ± 0.9, 12.2 ± 1.0, and 12.2 ± 1.0 minutes, respectively (**Figure 1B**). Compared to the Vehicle group, BCAA, CB-L, CB-H, HK-CB-L, and HK-CB-H supplementation significantly increased treadmill endurance by 1.09-fold (*p* = 0.0087), 1.16-fold (*p* = 0.0047), 1.22-fold (*p* = 0.0002), 1.22-fold (*p* = 0.0002), and 1.22-fold (*p* = 0.0002), respectively. However, no significant differences in treadmill endurance performance were observed between the BCAA group and the CB-L, CB-H, HK-CB-L, or HK-CB-H groups.

### Supplementation with Live or Heat-Killed *C. butyricum* GKB7 Improves Balance Performance in Mice with Low Innate Exercise Capacity

**Figure 2:**
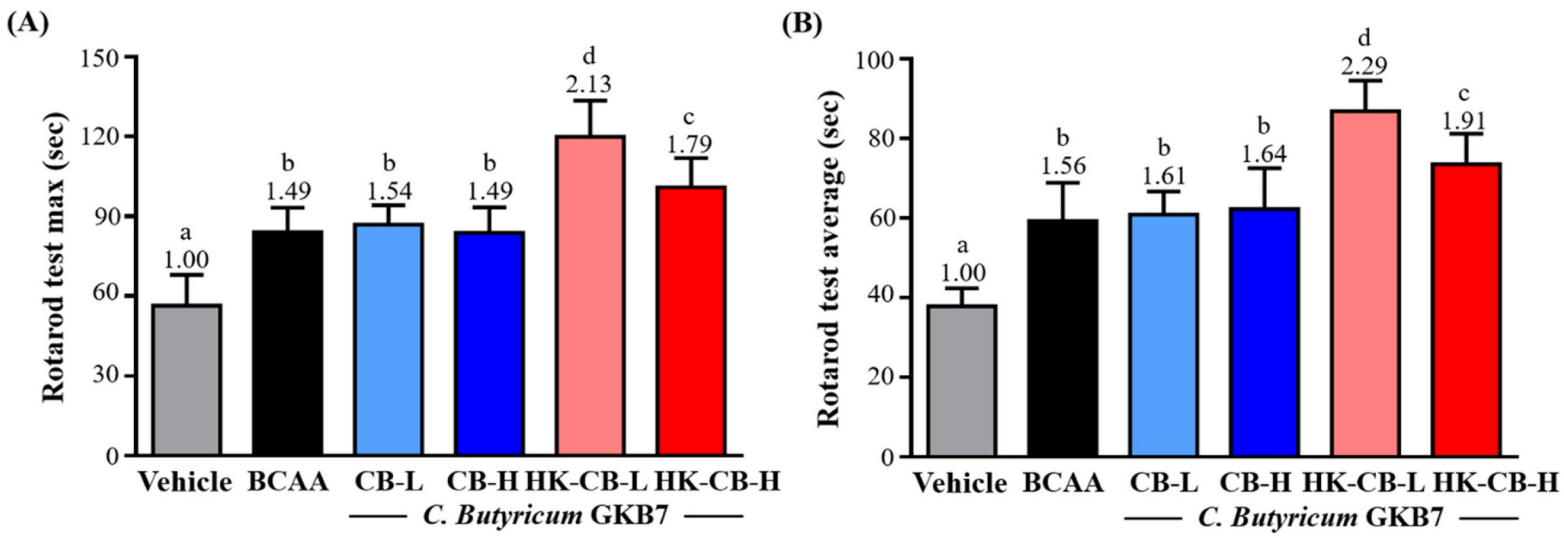
Effects of live or heat-killed *C. butyricum* GKB7 supplementation on balance performance. **(A)** Maximum balance performance and **(B)** average balance performance in each experimental group. The experimental animals were divided into six groups (n = 8 per group): Vehicle control group (Vehicle); Branched-chain amino acid control group (BCAA); Low-dose *C. butyricum* GKB7 group (CB-L); High-dose *C. butyricum* GKB7 group (CB-H); Low-dose heat-killed *C. butyricum* GKB7 group (HK-CB-L); High-dose heat-killed *C. butyricum* GKB7 group (HK-CB-H). All values are expressed as Mean ± SD. Different letters (a, b, c, d) indicate significant differences among groups, *P* < 0.05.

As shown in** Figure 2A**, the maximum balance time for the Vehicle, BCAA, CB-L, CB-H, HK-CB-L, and HK-CB-H groups was 56.4 ± 11.7, 84.0 ± 9.3, 86.9 ± 7.3, 83.8 ± 9.6, 119.9 ± 13.6, and 100.9 ± 11.0 seconds, respectively. Compared to the Vehicle group, supplementation with BCAA, CB-L, CB-H, HK-CB-L, and HK-CB-H significantly increased maximum balance time by 1.49-fold (*p* < 0.0001), 1.54-fold (*p* < 0.0001), 1.49-fold (*p* = 0.0009), 2.13-fold (*p* < 0.0001), and 1.79-fold (*p* < 0.0001), respectively. Compared to the BCAA group, only the HK-CB-L and HK-CB-H groups showed significant improvements in maximum balance time, increasing by 2.13-fold (*p* < 0.0001) and 1.79-fold (*p* = 0.0094), respectively.

For average balance time performance, as shown in **Figure 2B**, the Vehicle, BCAA, CB-L, CB-H, HK-CB-L, and HK-CB-H groups exhibited average balance times of 37.9 ± 4.5, 59.3 ± 9.6, 60.9 ± 5.8, 62.3 ± 10.2, 86.9 ± 7.6, and 73.5 ± 7.7 seconds, respectively. Compared to the Vehicle group, supplementation with BCAA, CB-L, CB-H, HK-CB-L, and HK-CB-H significantly increased average balance time by 1.56-fold (*p* < 0.0001), 1.61-fold (*p* < 0.0001), 1.64-fold (*p* < 0.0001), 2.29-fold (*p* < 0.0001), and 1.94-fold (p < 0.0001), respectively. Compared to the BCAA group, only the HK-CB-L and HK-CB-H groups showed significant improvements in average balance time, increasing by 2.29-fold (p < 0.0001) and 1.94-fold (p = 0.0265), respectively.

### Effects of Live or Heat-Killed *C. butyricum* GKB7 Supplementation on Glycogen Content in the Liver and Skeletal Muscle

To evaluate whether supplementation with low or high doses of live or heat-killed *C. butyricum* GKB7 (CB) enhances the storage of glycogen, a critical energy reserve, in body tissues, mice were sacrificed 30 minutes after the final supplementation at the end of the experiment. Liver and hind limb muscle tissues were collected for glycogen content analysis.

**Figure 3:**
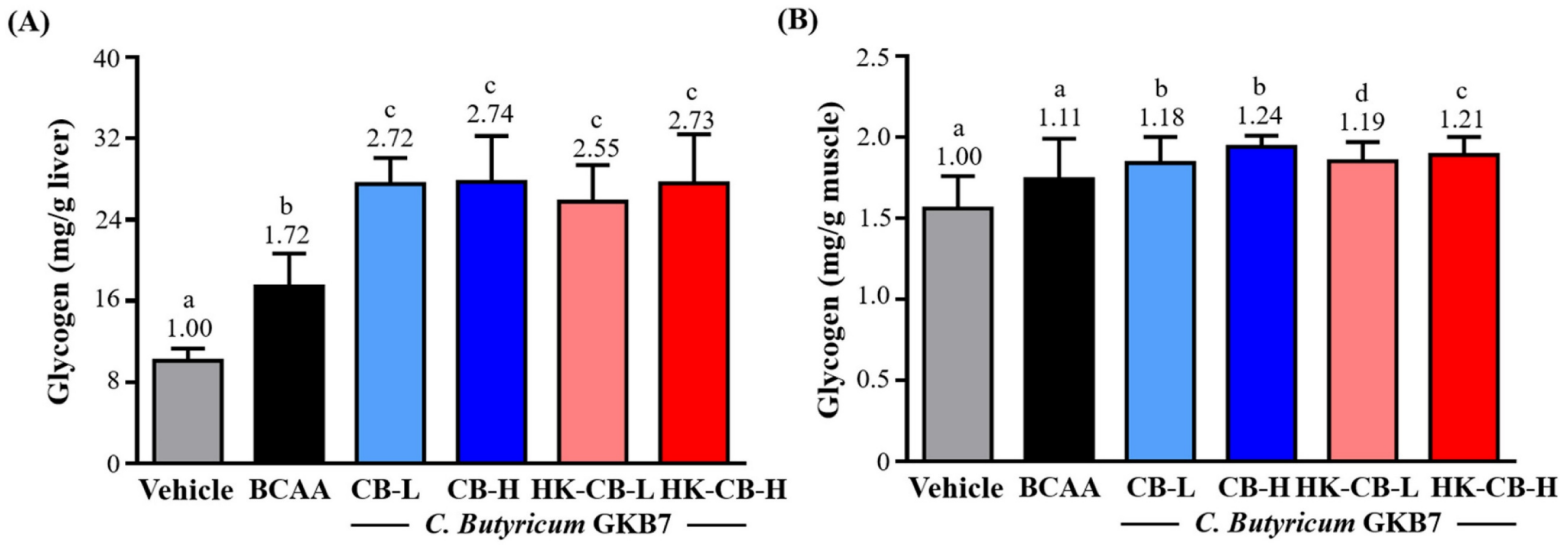
Effects of live or heat-killed *C. butyricum* GKB7 supplementation on **(A)** hepatic glycogen content and **(B)** skeletal muscle glycogen content. The experimental animals were divided into six groups (n = 8 per group): Vehicle control group (Vehicle); Branched-chain amino acid control group (BCAA); Low-dose *C. butyricum* GKB7 group (CB-L); High-dose *C. butyricum* GKB7 group (CB-H); Low-dose heat-killed *C. butyricum* GKB7 group (HK-CB-L); High-dose heat-killed *C. butyricum* GKB7 group (HK-CB-H). All values are expressed as Mean ± SD. Different letters (a, b, c, d) indicate significant differences among groups, *P* < 0.05.

Hepatic glycogen content: As shown in **Figure 3A**, the glycogen content in the liver for the Vehicle, BCAA, CB-L, CB-H, HK-CB-L, and HK-CB-H groups was 10.11 ± 1.21, 17.44 ± 3.24, 27.53 ± 2.55, 27.72 ± 4.53, 25.80 ± 3.60, and 27.58 ± 4.85 mg/g liver, respectively. Compared to the Vehicle group, hepatic glycogen content was significantly increased by 1.72-fold (*p* < 0.0001), 2.72-fold (*p* < 0.0001), 2.74-fold (*p* < 0.0001), 2.55-fold (*p* < 0.0001), and 2.73-fold (*p* < 0.0001) in the BCAA, CB-L, CB-H, HK-CB-L, and HK-CB-H groups, respectively. Compared to the BCAA group, hepatic glycogen content was significantly increased by 1.58-fold (*p* = 0.0007), 1.59-fold (*p* = 0.0218), 2.55-fold (*p* < 0.0001), and 2.73-fold (*p* < 0.0001) in the CB-L, CB-H, HK-CB-L, and HK-CB-H groups, respectively.

Skeletal muscle glycogen content: As shown in **Figure 3B**, the glycogen content in skeletal muscle for the Vehicle, BCAA, CB-L, CB-H, HK-CB-L, and HK-CB-H groups was 1.56 ± 0.20, 1.74 ± 0.25, 1.84 ± 0.16, 1.94 ± 0.07, 1.85 ± 0.12, and 1.89 ± 0.11 mg/g muscle, respectively. Compared to the Vehicle group, muscle glycogen content was significantly increased by 1.18-fold (*p* = 0.0014), 1.24-fold (*p* < 0.0001), 1.19-fold (*p* = 0.0010), and 1.21-fold (*p* = 0.0003) in the CB-L, CB-H, HK-CB-L, and HK-CB-H groups, respectively. Compared to the BCAA group, muscle glycogen content was significantly increased by 1.06-fold (*p* = 0.0406), 1.11-fold (*p* = 0.0024), 1.19-fold (*p* = 0.0319), and 1.21-fold (*p* = 0.0110) in the CB-L, CB-H, HK-CB-L, and HK-CB-H groups, respectively.

### Effects of Live or Heat-Killed C. butyricum GKB7 Supplementation on Oral Glucose Tolerance Test (OGTT)

**Figure 4:**
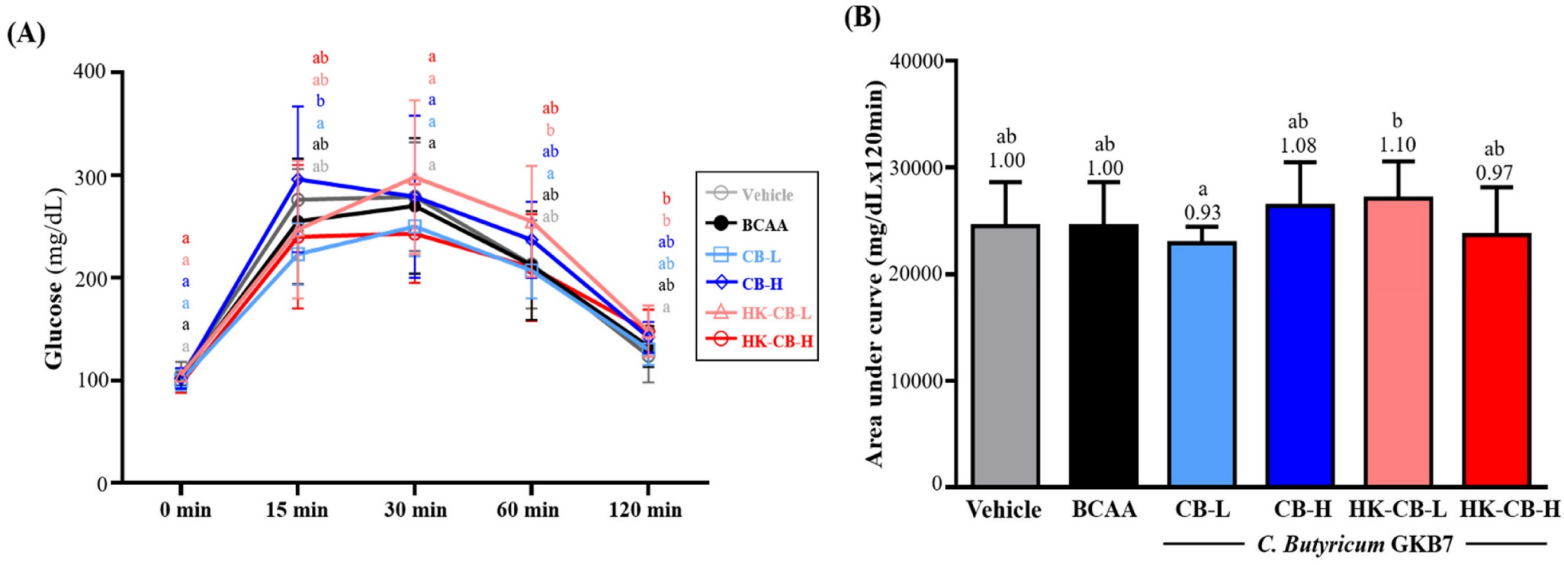
Effects of live or heat-killed *C. butyricum* GKB7 supplementation on **(A)** blood glucose changes and **(B)** area under the curve (AUC) during the oral glucose tolerance test. The experimental animals were divided into six groups (n = 8 per group): Vehicle control group (Vehicle); Branched-chain amino acid control group (BCAA); Low-dose *C. butyricum* GKB7 group (CB-L); High-dose *C. butyricum* GKB7 group (CB-H); Low-dose heat-killed *C. butyricum* GKB7 group (HK-CB-L); High-dose heat-killed *C. butyricum* GKB7 group (HK-CB-H). All values are expressed as Mean ± SD. Different letters (a, b) indicate significant differences among groups, *P* < 0.05.

As shown in **Figure 4A**, fasting blood glucose levels before the OGTT in the Vehicle, BCAA, CB-L, CB-H, HK-CB-L, and HK-CB-H groups were 105 ± 13, 103 ± 8, 100 ± 10, 102 ± 10, 105 ± 3, and 97 ± 9 mg/dL, respectively, with no significant differences among groups. Following glucose administration, blood glucose levels at different time points were as follows: At 15 minutes, blood glucose levels were 276 ± 30, 255 ± 61, 223 ± 30, 296 ± 71, 247 ± 67, and 240 ± 70 mg/dL for the Vehicle, BCAA, CB-L, CB-H, HK-CB-L, and HK-CB-H groups, respectively. No significant differences were observed between the CB-L, CB-H, HK-CB-L, and HK-CB-H groups compared to the Vehicle or BCAA groups. At 30 minutes, blood glucose levels were 279 ± 53, 270 ± 66, 250 ± 29, 279 ± 79, 298 ± 75, and 243 ± 48 mg/dL for the Vehicle, BCAA, CB-L, CB-H, HK-CB-L, and HK-CB-H groups, respectively, with no significant differences among groups. At 60 minutes, blood glucose levels were 213 ± 43, 212 ± 53, 207 ± 27, 237 ± 37, 255 ± 54, and 210 ± 52 mg/dL for the Vehicle, BCAA, CB-L, CB-H, HK-CB-L, and HK-CB-H groups, respectively. Again, no significant differences were observed among the experimental groups. At 120 minutes, blood glucose levels were 124 ± 26, 133 ± 20, 130 ± 15, 142 ± 15, 148 ± 25, and 148 ± 21 mg/dL for the Vehicle, BCAA, CB-L, CB-H, HK-CB-L, and HK-CB-H groups, respectively, with no significant differences among groups.

For the area under the curve (AUC) analysis of glucose response over 120 minutes (**Figure 4B**), the AUC values were 24,509 ± 3,930, 24,506 ± 4,146, 22,913 ± 1,525, 26,420 ± 4,092, 27,078 ± 3,514, and 23,686 ± 4,448 mg/dL × 120 min for the Vehicle, BCAA, CB-L, CB-H, HK-CB-L, and HK-CB-H groups, respectively. No significant differences in AUC values were found between the CB-L, CB-H, HK-CB-L, and HK-CB-H groups compared to the Vehicle or BCAA groups.

### Effects of Live or heat-killed *C. butyricum* GKB7 supplementation on blood biochemical parameters

As shown in **Table 2**, no significant differences were observed among the Vehicle, BCAA, CB-L, CB-H, HK-CB-L, and HK-CB-H groups for the following biochemical markers: Liver function markers: Aspartate aminotransferase (AST) and alanine aminotransferase (ALT). Renal function markers: Blood urea nitrogen (BUN), creatinine (CREA), and uric acid (UA). Lipid profile markers: Total cholesterol (TC), triglycerides (TG), high-density lipoprotein cholesterol (HDL), and low-density lipoprotein cholesterol (LDL). Other metabolic markers: Blood glucose (GLU) and creatine kinase (CK). These results indicate that supplementation with live or heat-killed *C. butyricum* GKB7 does not induce significant alterations in liver function, renal function, lipid metabolism, or glucose metabolism markers.

### Effects of Live or Heat-Killed *C. butyricum* GKB7 Supplementation on Tissue Organ Weight and Histological Analysis

Tissue and Organ Weights: As shown in **Table 3**, no significant differences were observed in liver and muscle tissue weights among the Vehicle, BCAA, CB-L, CB-H, HK-CB-L, and HK-CB-H groups. However, the epididymal fat pad weight in the HK-CB-L group was significantly reduced by 33.45% (*p* = 0.0053) compared to the BCAA group.

Since tissue and organ weights may be influenced by body weight variations, the relative organ weight (organ weight normalized to body weight) was also analyzed. No significant differences were found in the relative liver and muscle weights among groups. However, similar to the absolute weight, the relative epididymal fat pad weight in the HK-CB-L group was significantly reduced by 32.67% (*p* = 0.0066) compared to the BCAA group.

**Figure 5:**
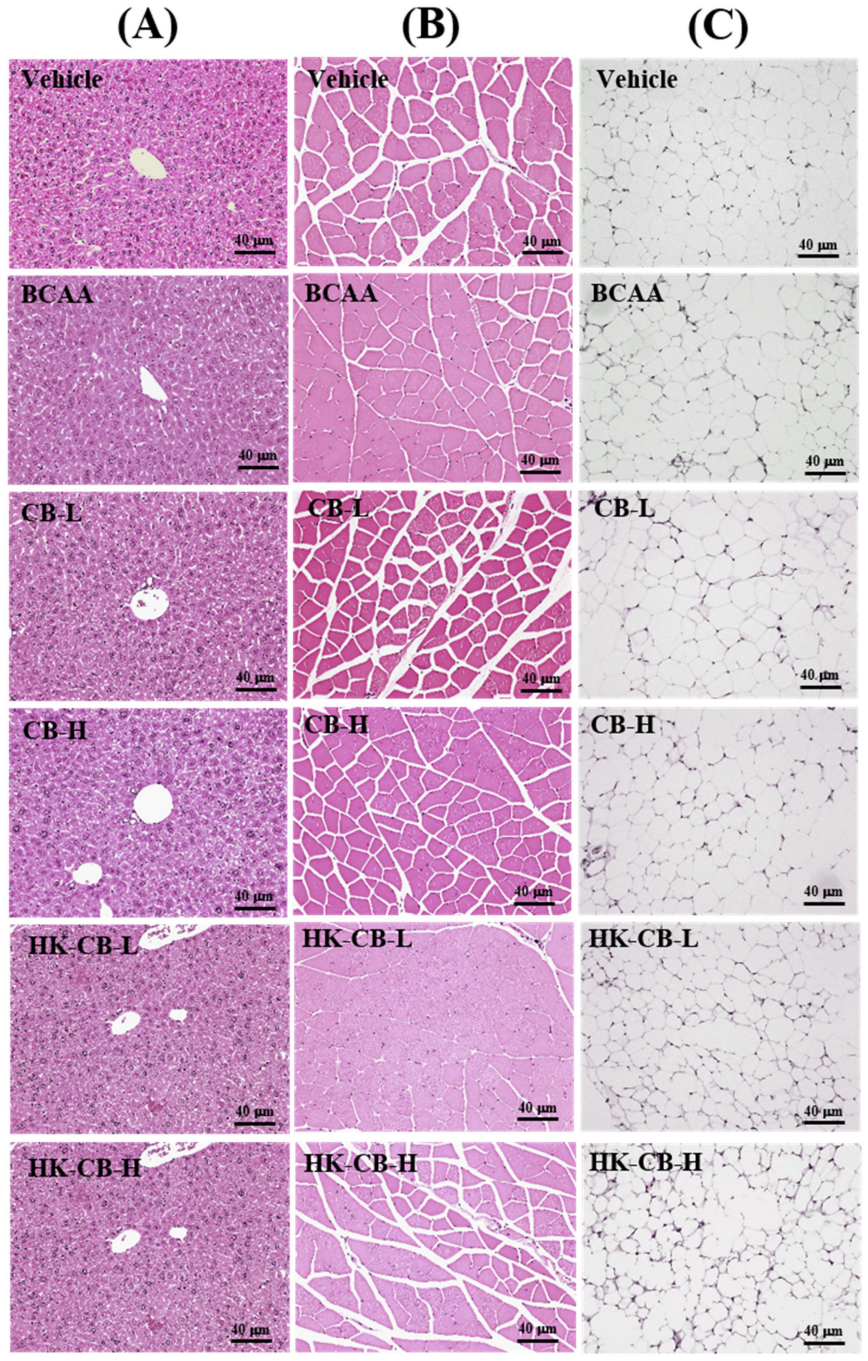
Histological analysis of tissue and organs following live or heat-killed C. butyricum GKB7 supplementation. The experimental animals were divided into six groups (n = 8 per group): Vehicle control group (Vehicle); Branched-chain amino acid control group (BCAA); Low-dose C. butyricum GKB7 group (CB-L); High-dose C. butyricum GKB7 group (CB-H); Low-dose heat-killed C. butyricum GKB7 group (HK-CB-L); High-dose heat-killed C. butyricum GKB7 group (HK-CB-H). Representative histological sections of (A) Liver, (B) Muscle, and (C) Epididymal fat pad (EFP) from each experimental group.

Histological Analysis: To assess whether four weeks of supplementation caused any tissue damage or pathological changes, histological analysis was conducted. As shown in **Figure 5**, no visible abnormalities, structural damage, or adverse effects were observed in the liver, muscle, or epididymal fat tissues across all experimental groups.

### Effects of Live or Heat-Killed *C. butyricum* GKB7 Supplementation on Tissue Organ Weight and Histological Analysis

**Figure 6:**
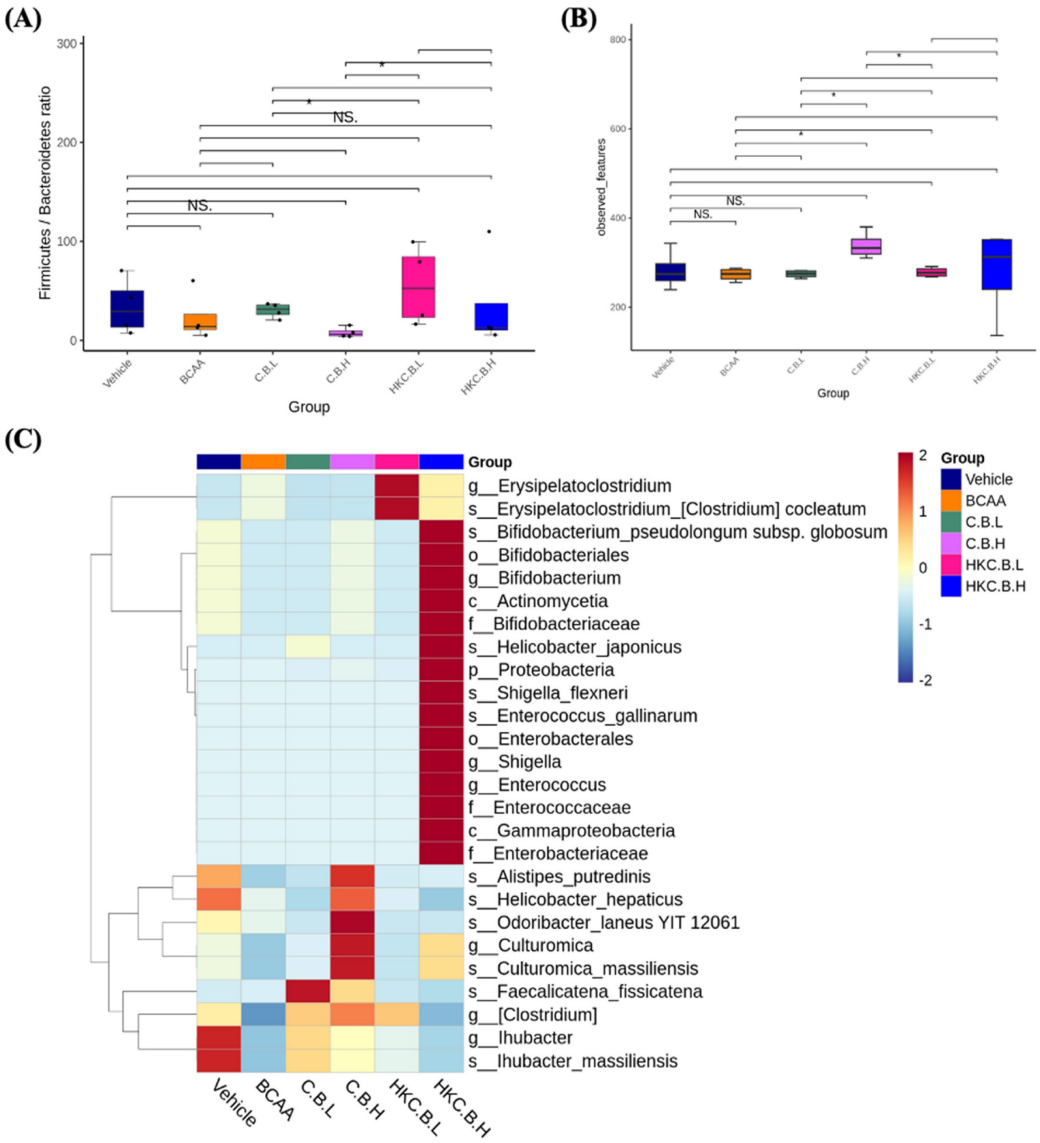
Effects of live or heat-killed *C. butyricum* GKB7 supplementation on gut microbiota composition. **(A)** Firmicutes/Bacteroidetes (F/B) ratio,** (B)** Alpha diversity indices, and **(C)** LEfSe heat map analysis of gut microbiota. The experimental animals were divided into six groups (n = 8 per group): Vehicle control group (Vehicle); Branched-chain amino acid control group (BCAA); Low-dose *C. butyricum* GKB7 group (CB-L); High-dose *C. butyricum* GKB7 group (CB-H); Low-dose heat-killed *C. butyricum* GKB7 group (HK-CB-L); High-dose heat-killed *C. butyricum* GKB7 group (HK-CB-H).

The Firmicutes/Bacteroidetes (F/B) ratio has been recognized as a key microbial factor in recent microbiome studies, often associated with disease risk. As shown in **Figure 6A**, the F/B ratio in the CB-L group was significantly lower than that in the HK-CB-L group (*p* < 0.05), and the CB-H group had a significantly lower F/B ratio than the HK-CB-H group (*p* < 0.05). These findings suggest that supplementation with live CB may contribute to a higher relative abundance of Bacteroidetes.

To assess gut microbial diversity, alpha diversity indices were analyzed. As shown in **Figure 6B**, observed species richness was significantly higher in the CB-H group compared to the CB-L group. Additionally, the HK-CB-L group exhibited a significant increase in microbial diversity compared to both the CB-L and CB-H groups, as well as the BCAA group.

To identify species with significantly different abundances across groups, LEfSe (Linear Discriminant Analysis Effect Size) analysis was performed, and the relative abundances of biomarkers were visualized as hierarchical heatmaps based on both individual samples and group means. As shown in **Figure 6C**, different dosages and forms of CB supplementation (live or heat-killed) led to distinct shifts in the relative abundances of specific bacterial taxa. Notably, most of the taxa enriched following CB supplementation were dominant or symbiotic beneficial bacteria, suggesting a potential positive impact on gut microbial composition.

## Discussion

The present study demonstrated that four weeks of supplementation with either live or heat-killed *C. butyricum* GKB7 (CB) significantly enhanced aerobic exercise endurance, balance performance, glycogen storage, and gut microbiota composition in mice with inherently low aerobic capacity. These findings provide compelling evidence that CB supplementation may serve as a potential ergogenic aid for improving physical performance and metabolic health in individuals with compromised exercise capacity.

The results from the weight-bearing swimming and treadmill exhaustion tests revealed that both live and heat-killed CB supplementation significantly increased endurance performance compared to the control and BCAA groups. The increase in endurance performance can be attributed to multiple factors, including enhanced energy metabolism, improved mitochondrial function, and reduced oxidative stress 16. Previous studies have indicated that gut microbiota-derived short-chain fatty acids (SCFAs), particularly butyrate, play a crucial role in enhancing mitochondrial efficiency and energy production 6. Butyrate has been shown to act as a substrate for mitochondrial oxidation, thereby increasing ATP production and sustaining prolonged exercise 17. This mechanistic insight aligns with our findings that CB supplementation, particularly at high doses, resulted in significantly improved aerobic performance.

Balance and coordination are critical factors influencing overall physical performance, particularly in activities requiring neuromuscular control 18. In our study, CB supplementation significantly improved balance performance in the rotarod test, with the heat-killed CB groups (HK-CB-L and HK-CB-H) showing the most pronounced effects. This suggests that CB's benefits extend beyond metabolic enhancement to neuromuscular function. Previous studies have linked SCFA production by gut microbiota to improved neuromuscular signaling and motor coordination through the gut-brain axis 7. It is plausible that CB supplementation modulated neural signaling pathways that contribute to motor function, though further mechanistic studies are needed to confirm this hypothesis.

Glycogen is a key energy reserve for sustaining prolonged exercise 19, and our results demonstrated that both live and heat-killed CB supplementation significantly increased glycogen storage in liver and skeletal muscle tissues. The ability of CB to enhance glycogen storage may be attributed to its role in modulating glucose metabolism and insulin sensitivity. Studies have shown that butyrate improves insulin signaling and enhances glucose uptake by skeletal muscle, leading to increased glycogen synthesis 20. The observed increase in hepatic glycogen content further supports the hypothesis that CB supplementation enhances systemic glucose homeostasis, providing an additional energy reservoir for endurance activities.

Despite the positive effects on glycogen storage, CB supplementation did not significantly alter glucose tolerance as measured by the oral glucose tolerance test (OGTT). While some previous studies have suggested that probiotic supplementation can modulate glucose metabolism 21, our findings indicate that CB's primary effects may be centered on energy storage rather than acute glucose clearance. It is possible that CB-induced metabolic adaptations require a longer intervention period or higher doses to manifest significant improvements in glucose tolerance. Further investigations are needed to explore potential dose-dependent effects and long-term metabolic adaptations following CB supplementation.

The gut microbiota plays a crucial role in regulating host metabolism, immune function, and exercise performance 22. Our study found that CB supplementation significantly altered gut microbial composition, with notable increases in the abundance of *Bacteroidetes* and overall microbial diversity. The decrease in the Firmicutes/Bacteroidetes (F/B) ratio observed in the CB-L and CB-H groups suggests a shift towards a more metabolically favorable microbiome profile. Previous studies have associated a lower F/B ratio with improved metabolic health and enhanced SCFA production, which could contribute to the observed improvements in endurance performance and glycogen storage 5.

Furthermore, the increased microbial diversity observed in the CB-H and HK-CB-H groups suggests that CB supplementation promotes a more resilient and functionally diverse gut microbiota. This is particularly important given the growing body of evidence linking microbial diversity to improved exercise adaptation and overall health 23. The specific taxa enriched following CB supplementation warrant further investigation to identify key microbial species contributing to these beneficial effects.

Interestingly, our results indicate that both live and heat-killed CB supplementation provided comparable benefits in terms of endurance performance, glycogen storage, and gut microbiota modulation. This suggests that the bioactive components of CB, such as SCFAs and microbial metabolites, may be responsible for the observed effects rather than the viability of the bacterial cells. Heat-killed probiotics have gained increasing attention for their ability to confer health benefits without the risks associated with live bacterial colonization. The significant improvements observed in the HK-CB groups suggest that non-viable bacterial components, such as cell wall-derived peptidoglycans and lipoteichoic acids, may play a role in modulating host metabolism and immune responses.

While this study provides valuable insights into the ergogenic and metabolic effects of CB supplementation, several limitations should be acknowledged. First, the exact molecular mechanisms underlying CB's effects on exercise performance and metabolic health remain unclear. Future studies should investigate the role of specific microbial metabolites, such as butyrate and propionate, in mediating these effects. Second, the study was conducted in a rodent model with inherently low aerobic capacity; thus, extrapolation to human populations requires further validation. Clinical trials assessing CB's efficacy in athletes and sedentary individuals could provide translational relevance. Lastly, the potential long-term effects of CB supplementation on systemic metabolism and immune function warrant further investigation.

## Conclusions

In summary, this study demonstrated that four weeks of supplementation with either live or heat-killed *Clostridium butyricum* GKB7 significantly improved aerobic endurance, balance performance, and glycogen storage while promoting a favorable gut microbiota composition in mice with low innate exercise capacity. These findings highlight the potential of CB as an ergogenic aid for individuals with impaired exercise performance. Future research should focus on elucidating the precise mechanisms underlying CB's beneficial effects and exploring its applications in human populations.

## Figures and Tables

**Table 1:** Changes in body weight and food intake during the experimental period

Characteristic	Vehicle	BCAA	*C. butyricum* GKB7			
			CB-L	CB-H	HK-CB-L	HK-CB-H
Initial BW (g)	37.5±1.8	37.8±1.3	37.8±1.3	37.4±1.5	37.4±1.1	37.2±1.0
1st wk BW	37.9±1.9	38.3±1.2	38.3±1.4	37.8±1.5	37.8±0.9	37.8±0.9
2nd wk BW	38.3±1.8	38.9±1.1	38.7±1.4	38.3±1.7	38.2±1.1	38.3±1.1
3rd wk BW	39.0±1.2	39.3±1.3	39.1±1.5	38.8±1.9	38.6±1.5	38.8±1.0
4th wk BW	39.5±1.3	39.7±1.3	39.6±1.6	39.4±2.1	39.1±1.8	39.5±1.2
5th wk BW	39.7±1.5	40.1±1.4	40.0±1.6	39.9±2.0	39.5±1.7	39.7±1.2
Final BW (g)	40.2±1.6	40.2±1.6	40.8±1.7	40.2±2.1	40.2±1.7	40.4±1.4
Water intake (mL/mouse/day)	8.55±0.74	8.50±0.69	8.54±0.68	8.48±0.79	8.48±0.89	8.56±0.76
Diet intake (g/mouse/day)	6.70±0.44	6.74±0.43	6.74±0.45	6.76±0.57	6.75±0.62	6.72±0.57

The experimental animals were divided into six groups (n = 8 per group): Vehicle control group (Vehicle); Branched-chain amino acid control group (BCAA); Low-dose *C. butyricum* GKB7 group (CB-L); High-dose *C. butyricum* GKB7 group (CB-H); Low-dose heat-killed *C. butyricum* GKB7 group (HK-CB-L); High-dose heat-killed *C. butyricum* GKB7 group (HK-CB-H). All values are expressed as Mean ± SD.

**Table 2:** Effects of live or heat-killed* C. butyricum* GKB7 supplementation on blood biochemical parameters at the end of the experiment

Parameters	Vehicle	BCAA	*C. butyricum* GKB7			
			CB-L	CB-H	HK-CB-L	HK-CB-H
AST(U/L)	76±8	77±6	77±6	77±6	77±9	76±10
ALT(U/L)	56±7	55±5	54±6	55±5	55±8	54±6
BUN (g/dL)	26.9±3.1	26.8±3.2	26.3±2.2	26.4±1.9	26.1±3.6	26.2±3.4
CREA (mg/dL)	0.36±0.02	0.36±0.01	0.35±0.01	0.36±0.02	0.36±0.02	0.36±0.02
UA (mg/dL)	1.8±0.4	1.8±0.3	1.8±0.3	1.8±0.3	1.8±0.5	1.8±0.4
TC (mg/dL)	161±11	159±11	160±7	161±5	162±4	160±10
TG (mg/dL)	179±9	179±9	180±11	179±12	180±11	179±7
HDL (mg/dL)	110.9±3.7	111.2±4.5	110.4±2.5	112.8±6.7	112.7±4.5	111.5±5.4
LDL (mg/dL)	24.2± 3.9	24.8±3.9	24.4±3.2	24.5±3.9	24.5±3.1	24.3±4.0
GLU (mg/dL)	229±20	230±20	226±21	229±25	228±16	230±15
CK (U/L)	179±23	180±14	179±20	179±18	180±17	179±14

The experimental animals were divided into six groups (n = 8 per group): Vehicle control group (Vehicle); Branched-chain amino acid control group (BCAA); Low-dose *C. butyricum* GKB7 group (CB-L); High-dose *C. butyricum* GKB7 group (CB-H); Low-dose heat-killed *C. butyricum* GKB7 group (HK-CB-L); High-dose heat-killed *C. butyricum* GKB7 group (HK-CB-H). All values are expressed as Mean ± SD.

**Table 3:** Effects of live or heat-killed *C. butyricum* GKB7 supplementation on tissue and organ weights

Characteristic	Vehicle	BCAA	*C. butyricum* GKB7			
			CB-L	CB-H	HK-CB-L	HK-CB-H
Liver (g)	2.38±0.24	2.38±0.23	2.35±0.30	2.39±0.19	2.40±0.25	2.36±0.23
Muscle (g)	0.39±0.03	0.40±0.02	0.38±0.04	0.39±0.03	0.39±0.03	0.38±0.02
EFP (g)	0.31±0.05^ ab^	0.37±0.10^ b^	0.32±0.06^ ab^	0.31±0.1^ab^	0.25±0.07^ a^	0.31±0.11^ ab^
**Relative**						
Liver (%)	5.90±0.40	5.83±0.42	5.78±0.82	5.88±0.35	5.99±0.75	5.85±0.53
Muscle (%)	0.96±0.06	0.97±0.04	0.92±0.10	0.97±0.08	0.97±0.10	0.93±0.07
EFP (%)	0.77±0.11^ ab^	0.91±0.24^ b^	0.78±0.14^ ab^	0.76±0.24 ^ab^	0.61±0.14^a^	0.77±0.29^ab^

The experimental animals were divided into six groups (n = 8 per group): Vehicle control group (Vehicle); Branched-chain amino acid control group (BCAA); Low-dose C. butyricum GKB7 group (CB-L); High-dose C. butyricum GKB7 group (CB-H); Low-dose heat-killed C. butyricum GKB7 group (HK-CB-L); High-dose heat-killed C. butyricum GKB7 group (HK-CB-H). All values are expressed as Mean ± SD. Values within the same row with different superscript letters (a, b) indicate significant differences (*P* < 0.05).
